# SPONGY (SPam ONtoloGY): Email Classification Using Two-Level Dynamic Ontology

**DOI:** 10.1155/2014/414583

**Published:** 2014-08-31

**Authors:** Seongwook Youn

**Affiliations:** Computer Science Department, University of Southern California, 941 Bloom Walk, SAL 300, Los Angeles, CA 90089-0781, USA

## Abstract

Email is one of common communication methods between people on the Internet. However, the increase of email misuse/abuse has resulted in an increasing volume of spam emails over recent years. An experimental system has been designed and implemented with the hypothesis that this method would outperform existing techniques, and the experimental results showed that indeed the proposed ontology-based approach improves spam filtering accuracy significantly. In this paper, two levels of ontology spam filters were implemented: a first level global ontology filter and a second level user-customized ontology filter. The use of the global ontology filter showed about 91% of spam filtered, which is comparable with other methods. The user-customized ontology filter was created based on the specific user's background as well as the filtering mechanism used in the global ontology filter creation. The main contributions of the paper are (1) to introduce an ontology-based multilevel filtering technique that uses both a global ontology and an individual filter for each user to increase spam filtering accuracy and (2) to create a spam filter in the form of ontology, which is user-customized, scalable, and modularized, so that it can be embedded to many other systems for better performance.

## 1. Introduction

Email has been an efficient and popular communication mechanism as the number of Internet users increases. Therefore, email management has become an important and growing problem for individuals and organizations because it is prone to misuse. The blind posting of unsolicited email messages, known as spam, is an example of misuse. Spam is commonly defined as sending of unsolicited bulk email—that is, email that was not asked for by multiple recipients. A further common definition of spam is restricted to unsolicited commercial email, a definition that does not include noncommercial solicitations such as political or religious pitches, even if unsolicited, as spam. Email was by far the most common form of spamming on the Internet. In Q2 2013, it was reported that more than 70% of all emails sent worldwide have been classified as spam [1].

Spam filters using the structure and syntax of an email body in accordance with training techniques are common [2]. Also, to solve the spam problem, many methodologies based on the Bayesian classification are suggested by researchers. International data group [3] expected that global email traffic surges to 60 billion messages daily. It involves sending identical or nearly identical unsolicited messages to a large number of recipients. Unlike legitimate commercial email, spam is generally sent without the explicit permission of the recipients and frequently contains various tricks to bypass email filters.

Modern computers generally come with some ability to send spam. The only necessary ingredient is the list of addresses to target. Spammers obtain email addresses by a number of means: harvesting addresses from Usenet postings, DNS listings, or Web pages, guessing common names at known domains (known as a dictionary attack), and “e-pending” or searching for email addresses corresponding to specific persons, such as residents with given in an area. Many spammers utilize programs called web spiders to find email addresses on web pages, although it is possible to fool the web spider by substituting the “@” symbol with another symbol, for example, “#,” while posting an email address. As a result, users have to waste their valuable time to delete spam email. Moreover, because spam emails can fill up the storage space of a file server quickly, they could cause a very severe problem for many websites with thousands of users.

Much work on spam email filtering has been done using techniques such as decision trees, Naive Bayesian classifiers, and neural networks. To address the problem of growing volumes of unsolicited email, many different methods for email filtering are being deployed in many commercial products. We constructed a framework for efficient email filtering using ontologies. Ontologies allow for machine-understandable semantics of data, so it can be used in any system [4–6]. It is important to share the information with each other for more effective spam filtering. Thus, it is necessary to build ontologies and a framework for efficient email filtering. Using ontologies that are specially designed to filter spam, most of unsolicited bulk email could be filtered out on the system. This paper proposes to find an efficient spam email filtering method using ontologies. We used waikato environment for knowledge analysis (Weka) explorer and Jena to make ontologies based on a sample dataset [7].

Email can be classified using different methods. Different people or email agents may maintain their own personal email classifiers and rules. The problem of spam filtering is not a new one and there are already a dozen of different approaches that have been implemented. The challenge was more specific to areas like artificial intelligence and machine learning. Several implementations had various trade-offs, different performance metrics, and different classification efficiencies. The techniques such as decision trees, naive Bayesian classifiers, and neural networks had various classification efficiencies. The remainder of the paper is organized as follows: Section 2 describes existing related works; Section 3 provides our spam filtering framework using ontologies; Section 4 discusses the experimental result of the framework that we proposed; Section 5 concludes the paper with possible directions for future work.

## 2. Related Work

A rule-based system was suggested to classify spam email, but specific terms caused the failure of filtering [8]. Traditionally, Naïve Bayesian classifier was very popular method for document, text, and email classification system [9]. Shankar et al. and Yang et al. developed an algorithm to reduce the feature space without sacrificing remarkable classification accuracy, but the effectiveness was based on the quality of the training dataset [10, 11]. Yang et al. demonstrated that the feasibility of the approach to find the best learning algorithm and the metadata to be used, which is a very significant contribution in email classification using the rainbow system [12].

Androutsopoulos et al. presented an investigation to construct effective antispam filter using four learning algorithms, Naïve Bayes, Flexible Bayes, LogitBoost, and SVM. They used n-gram and information (IG) to select features. To compare the several learning algorithms fairly, they constructed four benchmark corpora. Finally, they insisted that mixtures of different filtering approaches using machine learning mechanism are very efficient, so combining several learning algorithms is promising [8].

Aery and Chakravarthy proposed a graph based mining approach for email classification in which structures/patterns can be extracted from a preclassified email folder and the same approach can be used effectively for classifying incoming email messages [13]. Approaches to filtering junk email are considered in [14–16]. Fawcett and Hotho et al. showed approaches to filtering email involve the deployment of data mining techniques [17, 18]. Cui et al. proposed a model based on the Neural Network (NN) to classify personal email and the use of Principal Component Analysis (PCA) as a preprocessor of NN to reduce the data in terms of both dimensionality as well as size [19, 20]. Androutsopoulos et al. compared the performance of the Naïve Bayesian filter to an alternative memory based learning approach on spam filtering [8].

Gupta et al. proposed a way to overcome to certain limitations due to embedded obfuscation like complex backgrounds, compression artifacts and wide variety of fonts and formats. Their methodology consists of 4 steps (identification of noise, extraction of low level features or calculation of entropy, removal of noise, content extraction using OCR) [21]. Their method showed about an accuracy of 93.3%. Woods et al. tried to show that using the low level image feature-edge, as well as the magnitude of the edges per image, it is possible to analyze and classify an image as spam or ham. They employed the Sobel edge detection algorithm, which analyzes a low level feature of an image as an alternative to the OCR only based filtering system [22].

Mavroeidis et al. addressed the problem by proposing a Word Sense Disambiguation (WSD) approach based on the intuition that word proximity in the document implies proximity also in the Hierarchical Thesauri (HT) graph [23]. Bringing in other kinds of features, which are spam-specific features in their work, could improve the classification results. A good performance was obtained by reducing the classification error by discovering temporal relations in an email sequence in the form of temporal sequence patterns and embedding the discovered information into content-based learning methods [24]. Meyer and Whateley showed that the work on spam filtering using feature selection based on heuristics [25].

Cormack and Lynam tested several open-source spam filters. Their approach is different from others in that the test set is large, comprises uncensored raw messages, and is presented to each filter sequentially with incremental feedback. They insisted that the risk of loss depends on the nature of the message, and that messages seem to be lost may be those that are less important [26, 27].

Liu presented a technique to help various classifiers to improve the mining of category profiles. The technique helps to create dynamic category profiles with respect to a received document, and accordingly helps to make proper filtering and classification decisions [28].

Yang et al. presented a comparative study of five feature selection methods (document frequency, information gain, mutual information, a *χ* ^2^ test, and term strength) in statistical learning of text categorization. They used a Reuter corpus as a dataset, and both a k-nearnest neighbor classifier (kNN) and a Linear Least Squares Fit (LLSF) mapping. In their experiment, they found strong correlations between the document frequency, information gain, and *χ* ^2^ values of a term. Document Frequency can be reliably used as a feature selection method because it was the simplest method with the lowest cost in computation. Experimental results of Information Gain and *χ* ^2^ were much better than those of Mutual Information and Term Strength [12].

Forman presented an empirical comparison of twelve feature selection methods with several corpuses like Reuters, TREC, OHSUMED, and so forth. He suggested a new feature selection metric, which is binormal separation (BNS). BNS outperformed the other feature selection methods in his experiment. His experiment was performed using a several classifiers including Naïve Bayesian, C4.5, logistic regression and SVM with a linear kernel [29].

## 3. The SPONGY (SPam ONtoloGY) System

The procedure of the SPONGY system operation is as follows.

1. Training dataset is selected. Training dataset is a collection of text-oriented email data.
2. Features from the dataset are selected using* tfidf*.
3. Weka input file is created based on the selected features and the dataset. Weka is a toolkit of machine learning algorithms written in Java for data mining tasks.
4. Through Weka, classification results are generated.
5. The classified results are converted to RDF file.
6. The converted RDF file is fed into Jena, which is a Java framework for building Semantic Web applications. It provides a programmatic environment for RDF, RDFS, OWL, and SPARQL and includes a rule-based inference engine.
7. Using Jena, ontologies are created, and we can give a query to Jena. Jena will give an output for the query using ontologies created in Jena.

Through these procedures, global and user-customized ontology filters are created. Incorrectly classified emails through global ontology filter are inserted into the user-customized ontology filter.

In contrast to previous approaches, ontologies were used in our approach. In addition, the C4.5 decision tree was used to classify the training dataset. The ontologies created by the implementation are modular, so those could be used in another system. In our previous classification experiment, the C4.5 showed better results than naïve Bayesian, neural network, or support vector machine (SVM) classifier.

### 3.1. Spam Filtering Approach

An assumption to create decision trees would be the intelligence behind the classification, but this was not enough because the decision tree ultimately is not a true ontology and also querying a decision tree was not easy. Once we narrowed down the type of decision tree that we were going to use, the next step was to create ontology based on the classification result through the C4.5. The RDF which would be the form of “Subject-Object-Predicate” was used to create ontology. Hence, our second main assumption was that we will need to map the decision tree into a formal ontology and query this ontology using our test email to be classified as spam or not. The test email is another thing we needed to consider because first, it is very difficult to deploy our system in such a way that it could read an incoming mail on a mail server and this would require extra work which would make the work unnecessarily complicated.

Weka [7] was used in the system. Weka is an open source software package, which has been implemented in object-oriented Java class hierarchy. Weka provides powerful machine algorithms and classification algorithms for data mining tasks. Also, it provides association rules, clustering algorithms, and regression.

The initial step was to gather a good dataset on which the decision tree will be based. This data should include the characteristics of spam email as well as the nonspam email. Also the attributes and the values for each type of email must be such that the decision tree based on the training data will not be biased. We evaluated a number of implementations for the decision trees and decided to use the Weka explorer for implementation of C4.5 decision tree. The tree accepts input in an Attribute-Relation File Format (ARFF) format. ARFF files have two distinct sections. The first section is the header information, which is followed by the data information.

The Header of the ARFF file contains the name of the relation, a list of the attributes (the columns in the data), and their types. Each data instance is represented on a single line, with a carriage return denoting the end of the instance. Attribute values for each instance are delimited by commas. The order that was declared in the header section should be maintained (i.e., the data corresponding to the* n*th @attribute declaration is always the* n*th field of the attribute). Missing values are represented by a single question mark. The training dataset was converted to ARFF format. Based on the training dataset, a decision tree was formed. This decision tree is a type of ontology.

The above file is a sample ARFF file where the word next to @relation is the just a name. It could be the name of the file, and name. It just signifies a header. The word next to the @attribute is the feature element on the basis of which the classification is going be done and our tree is being built. The value next to it after the “:” is its type. The last attribute in this list must be the final classifier of what we are looking for. In this case, the final classification result should be “1” if it is finally spam, otherwise, it should be “0.” All the leaf nodes on the classification result should be “1” or “0.” It is a rule in the ARFF file that the last attribute be the final classification result needed. After the @data, a set of values which are values of the attributes will be placed.

 @relation spamchar
 @attribute word_freq_make: real
 @attribute word_freq_address: real
 @attribute word_freq_all: real
 @attribute word_freq_3d: real
 @attribute word_freq_our: real
 @attribute word_freq_over: real
 @attribute word_freq_remove: real
 @attribute word_freq_internet: real
 @attribute word_freq_order: real
 @attribute word_freq_mail: real
 @attribute ifspam {1,0}
 @data
 0,0.64,0.64,0,0.32,0,0,0,0,0,0
 0,0.67,0.23,0,0.17,0.6,1.6,0,1,0.9,1.

The number of values will equal the number of attributes and the order is such that the first value in the dataset corresponds to the first attribute.

For the First mail:

 word_freq_make is 0 and word_freq_all is 0.64.
 Similarly, for the Second mail:
 word_freq_make is 0 and word_freq_all is 0.23.

These values are calculated as follows:

100∗Number of words or characters in the attribute/total number of words in the email.

If you notice, in both the datasets, the last values are either 0 or 1 which means that this mail is should be classified as spam if 1 or not spam if 0.

### 3.2. SPONGY System Architecture

**Figure 1:**
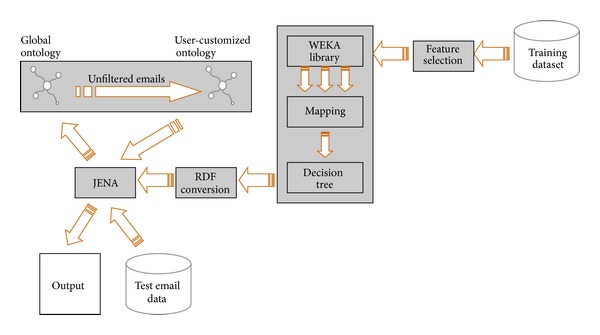
SPONGY architecture.

Figure 1 shows our framework to filter spam. It is named the SPONGY (SPam ONtoloGY) system. The training dataset is the set of email which gives us a classification result. The test data is actually the email will run through our system which we test to see if it is classified correctly as spam or not. This will be an ongoing test process and since the test data is not finite because of the learning procedure, the test data will sometimes merge with the training data. The training dataset was used as input to the C4.5 classification. To do that, the training dataset should be modified as a compatible input format. The SPONGY system gives us the classification result using the C4.5 classifier.

To query the test email in Jena, ontology should be created based on the classification result. To create the ontology, ontology language was required. RDF was used to create ontology. The classification result of the RDF format was input to Jena, and input RDF was deployed through Jena; finally, ontology was created. Ontology generated in the form of an RDF data model is the base on which the incoming mail is checked for its legitimacy. Depending upon the assertions that we can conclude from the outputs of Jena, the email can be defined as either spam or legitimate. The email is actually the email in the format that Jena will take in (i.e., in a CSV format) and will run through the ontology that will result in spam or legitimate.

The SPONGY system periodically updates the dataset with the emails classified as spam when user spam report is requested. Then, a modified training dataset is input to WEKA to get a new classification result. Based on the classification result, we can get a new ontology, which can be used as a second spam filter (that is user-customized ontology). Through this procedure, the number of ontologies will be increased. Finally, these spam filtering ontologies will be customized for each user. User customized ontology filter would be different from the other depending on each user's background, preference, hobby, and so forth. That means one email might be spam for person A but not for person B. User customized ontology evolves periodically and adaptively. The SPONGY system provides an evolving spam filter based on users' preferences, so users can get a better spam filtering result.

The input to the system is mainly the training dataset and then the test email. The test email is the first set of email that the system will classify and learn and after a certain time, the system will take a variety of email as input to be filtered as a spam or legitimate. For the training dataset we used, several feature selection algorithms including naïve Bayesian, neural network, SVM, and C4.5 were tested; the C4.5 and Naïve Bayesian classifiers showed good performance on the training email dataset [23]. The classification results through Weka need to be converted to ontology. The classification result which we obtained through the C4.5 decision tree was mapped into the RDF format. This was given as an input to Jena which then mapped the ontology for us. This ontology enabled us to decide the way different headers and the data inside the email are linked based upon the word frequencies of each word or character in the dataset. The mapping also enabled us to obtain assertions about the legitimacy and nonlegitimacy of the email. The next part was using this ontology to decide whether a new email is a spam or legitimate. This required querying of the obtained ontology which was again done through Jena. The output obtained after querying was the decision whether the new email is a spam or legitimate. In summary, test email is checked whether it is spam or legitimate based on global ontology created with training dataset and mis-filtered emails are checked again based on a user-customized ontology created with user's spam report. With the help of adaptive user customized ontology, total spam filtering rate (the correct classification percentage) will be increased.

The primary way in which a user can let the system know would be through a GUI or a command line input with a simple “yes” or “no.” This would all be a part of a full-fledged working system as opposed to our prototype, which is a basic research experimental system.

### 3.3. SPONGY System Implementation

In the experiment,* tfidf* is selected as a feature selection algorithm for the experiment.* tfidf* is a popular text processing method for dealing with the textual features.

For the classification method, the C4.5 decision tree is used because it showed good performance compared with Neural Network, SVM, or naïve Bayesian classifier as we showed it our previous paper [5].

#### 3.3.1. Global Ontology Creation Procedure

Algorithm 1 is a pseudocode for global ontology filter creation in the SPONGY system.

#### 3.3.2. User-Customized Ontology Creation Procedure

For user-customized ontology, user profile ontology was used as shown in Algorithm 2. It was different with global ontology creation procedure. Using user profile ontology, preference of a specific user would be adapted in feature selection procedure. User profile ontology had a list of people to block their email and a list of words to block the emails related with some topic that is disliked by user. These blacklists and words will be combined with the words that were selected from the* tfidf*.

## 4. Experimental Results

In the initial SPONGY system, a global ontology was created with a 2108 email dataset (42.82% were spam and 57.18% were legitimate email). The* tfidf* mechanism was used as a feature selection algorithm. In the Weka, the C4.5 decision tree algorithm was used for email classification because the C4.5 showed the best result compared with Neural Network, Naïve Bayesian, and SVM. The classified result would be modified to RDF file format semi-automatically in the SPONGY system. The modified RDF file was entered into Jena, so the ontology was created for spam filtering. Finally, the test email data can be tested in the SPONGY system whether it is spam or not. After the SPONGY system was initialized, ontologies as a spam filter would be evolved adaptively on users' spam report.

The time complexity of ontologies creation is *O*(*mn*). *m* is the number of features and* n* is the size of dataset. However, ontologies are created off-line in a batch mode; the ontologies do not get updated in run-time. Also, the dataset collected to create user-customized ontology is from a single user only, hence the size of the dataset is relatively small. Therefore, time complexity is not a big issue for our system.

### 4.1. SPONGY System Results with Training Dataset for Global Ontology

Algorithm 3 shows how we choose the C4.5 classification filter, which uses the simple C4.5 decision tree for classification. Algorithm 3 shows that word “remove” was selected as a root node by the C4.5 classification.

Algorithm 4 shows the classification result including precision and recall. The confusion matrix shows the number of elements classified correctly and incorrectly as the percentage of classification.

**Figure 2:**
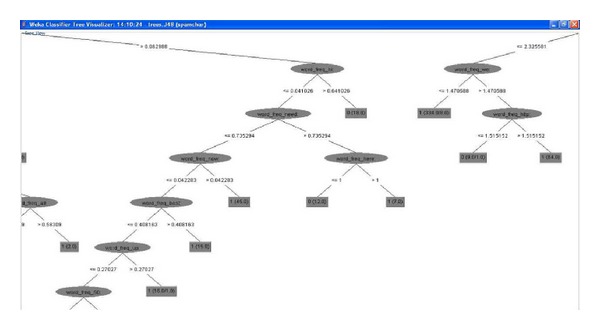
Tree of C4.5 classification result.

Figure 2 shows the classification result using the C4.5 classifier. The whole result is too big, so Figure 2 is just a part of it. In the leaf node, 1 means spam and 0 means legitimate.

**Figure 3:**
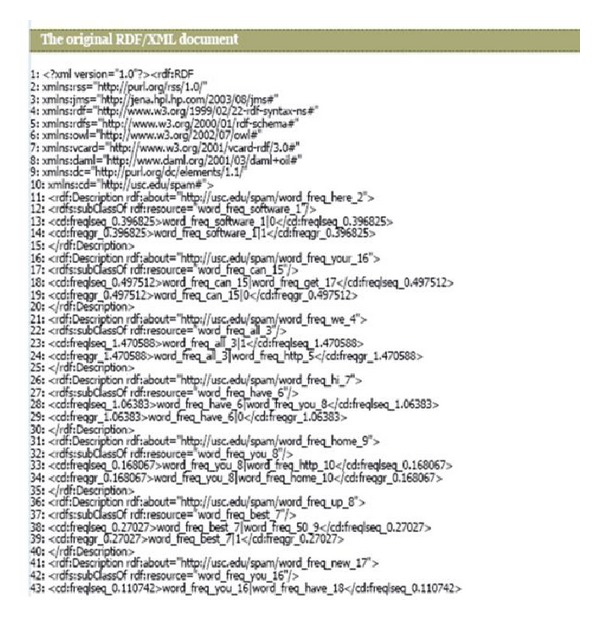
Converted RDF file of C4.5 classification result.

According to Figure 3 (based on the classification result of C4.5), for example, if the normalized value of the word “people” is greater than 0.18, email is classified as legitimate, otherwise, the system will check the normalized value of the word “our.” Finally, if the normalized value of the word “mail” is greater than 0.24, then the email is classified as spam. This result is from my experiment. Different classification results would be given if the experiment was performed with different dataset. The ontology using RDF was created based on the classification result. Figure 3 shows the RDF file created based on the C4.5 classification result. The RDF file was used as an input to Jena to create ontology which will be used to check if the test email is spam or not.

**Figure 4:**
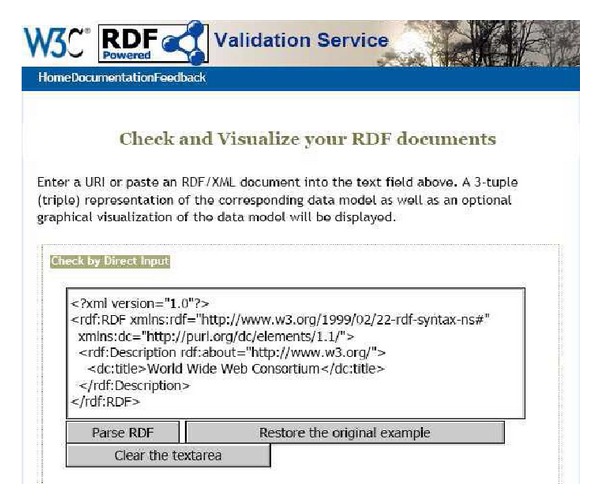
W3C RDF validation service.

Figure 4 shows RDF validation services. W3C RDF validation to give as input to Jena is syntactically correct or not. Because the RDF file based on the classification result using the C4.5 was created by us, and should be compatible with Jena, the validation procedure for syntax validation was required.

**Figure 5:**
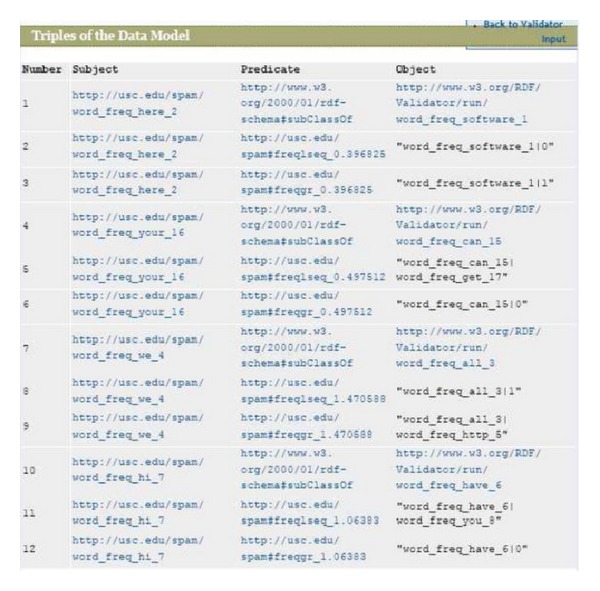
Triplets of RDF data model.

Figure 5 also shows the database of subject-predicate-object model we got after inputting the RDF file into Jena. This ontology model is also produced in Jena.

**Figure 6:**
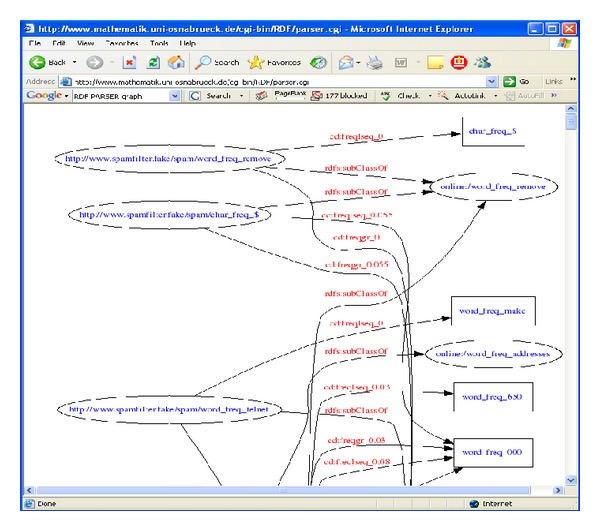
RDF data model (Ontology).

Figure 6 shows the RDF data model or ontology model. This model is obtained from the W3C validation schema. The ontology is obtained in Jena and not displayed directly. However, it can be shown using the graphics property of the Jena.

Total of 2108 emails were used as a training dataset. 47.8% of dataset were spam and 52.2% were legitimate email. The C4.5 classifier was used to classify the dataset in Weka explorer. 91.51% of emails were classified correctly and 8.49% were classified incorrectly. In the case of spam, precision was 0.872, recall was 0.963. In the case of legitimate email, precision was 0.962, recall was 0.871. The result is on Table 1. As in Figures 5 and 6, based on the C4.5 classification result, the ontology was created in the RDF format using Jena. This ontology was used to check input email through Jena.

The result was generated after we considered the word frequencies of various words inside the email and then querying our ontology data model for these word frequencies. If the value we get after comparing all the word frequencies of the email words is “0,” then the result is that the email is not spam and if the value is “1” then the result is that the email is spam. The result may have false positives (legitimate mail termed as not spam) or false negatives (spam email termed as not spam).

### 4.2. SPONGY System Results with User Dataset for User-Customized Ontology

In Section 3.1, global ontology filter was created based on a sample email dataset. Now the SPONGY system creates a user-customized adaptive ontology filter based on received emails of a specific user. Every user has different background and preference, so a user-customized adaptive ontology is different for each person. At first, emails of a user go through the global ontology filter, and the emails that are not classified correctly are entered into the user-customized adaptive ontology. The user-customized adaptive ontology will filter out the emails that are not classified correctly again. The user-customized adaptive ontology created by the SPONGY system gives us good performance improvement because the ontology filter was made based on specific user's email set. The initial global ontology already showed good performance.

At first, the experiment was performed with 200 emails (100 spam and 100 legitmate). The experimental result through the Weka is shown in Tables 2 and 3.

Then, we increased user dataset to 400 and then 600. The experiment was performed with 400 emails (200 spam and 200 legitmate). The experimental result through the Weka is shown in Tables 4 and 5.

Finally, the experiment was performed with 600 emails (300 spam and 300 legitimate). Most of the procedures to create user-customized ontology are similar to the case of global ontology creation except for the use of user profile ontology.

As shown on Tables 6 and 7, total of 600 emails were used as a specific user dataset. 50% of dataset was spam and 50% was legitimate email. The C4.5 classifier was used to classify the dataset in Weka explorer. 92.17% of emails were classified correctly and 7.83% were classified incorrectly. In the case of spam, precision was 0.882 and recall was 0.973. In the case of legitimate and precision was 0.970, recall was 0.870.

### 4.3. SPONGY System Results Using Both Global Ontology and User-Customized Ontology

Through the global ontology, 179 out of 2108 emails were classified incorrectly. In detail, 37 of 1008 spam emails and 142 of 1100 legitimate emails were classified incorrectly. These 179 (37 spam and 142 legitimate) emails were input to the user-customized ontology for filtering.

With the user-customized ontology created using 200 user emails, 10 of incorrectly classified 37 spam emails and 55 of incorrectly classified 142 legitimate emails were filtered out. In total, 114 of 2108 emails were classified incorrectly, but 179 of 2108 emails were classified incorrectly when we used only global ontology. The rate of correct classification is from 91.5085% to 94.5920%.

With the user-customized ontology created using 400 user email, 13 of incorrectly classified 37 spam emails and 57 of incorrectly classified 142 legitimate emails were filtered out. In total, 109 of 2108 emails were classified incorrectly, but 179 of 2108 emails were classified incorrectly when we used only global ontology. The rate of correct classification is from 91.5085% to 94.8292%.

With the user-customized ontology created using 600 user email, 14 of incorrectly classified 37 spam emails and 69 of incorrectly classified 142 legitimate emails were filtered out. In total, 96 of 2108 emails were classified incorrectly, but 179 of 2108 emails were classified incorrectly when we used only global ontology. As shown on Table 8, for the whole SPONGY system, in the case of spam email, precision was 0.931 and recall was 0.977 and, in the case of legitimate email, precision was 0.978 and recall was 0.934.

All the experimental results are summarized on Table 9. It registers much improvement. As shown on Table 9, when the first-level global ontology filter was used alone, 37 out of 1008 spam emails and 142 out of 1100 legitimate emails were classified incorrectly. However, in the case of using the second level user-customized ontology filter created from 600 user emails, 14 out of 37 incorrectly classified spam emails were saved and 69 out of 142 incorrectly classified legitimate emails were saved. As a result, correct classification percentage was increased from 91.5085% to 95.4459%.

### 4.4. Comparison with Other Spam Email Filters

Commercial spam filters supports many features as you can see in Table 10. Preset categories are provided by the program vendor freely. It contains content such as financial, adult content, and health. Rule customization option will allow you to add, remove, or modify the filtering rules. A rule is a set of criteria for determining whether or not an email is spam or legitimate.

However, most of commercial filters are too complicated and difficult for the end users. As you can see, most of filters can allow or block IP address, server, and email address. There are many other known filters in the world [30].

Generally, accuracy comparison among spam email filters should be done under the same dataset and same environment.

## 5. Conclusion and Discussion

In this paper, two levels of ontology spam filters were implemented: the global ontology filter and the user customized ontology filter.

The C4.5 decision tree algorithm, and so forth, showed about 91% of spam filtering rate, which is comparable with other authors' similar works. Additionally, a user-customized ontology filter was utilized as a second level filter. The user-customized ontology filter was created based on the specific user's background as well as the filtering mechanism used in the global ontology filter creation. Through a set of experiments, it was proven that the better spam filtering rate can be achieved using the user-customized ontology filter, which is adaptive and scalable. The same idea was adopted for the text-oriented email datasets, but it can be also used for other classification or clustering jobs.

Use of ontologies to help email classification is the important objective of the paper and those ontologies were successfully implemented. Learning motivation was that this approach has been taken and opens up a whole new aspect of email classification on the semantic web. Also, this approach fits into any system because the ontologies that were implemented in the paper are generic in nature. The technique introduced here will have a great advantage for systems ahead. As mentioned above, the classification accuracy can be increased initially by pruning the tree and using better classification algorithms, more number, and better classifiers or feature elements. These are bigger issues in the machine learning and artificial intelligence domain which are not primary concerns but helped in better classification after all.

The paper, as mentioned earlier, is more research-oriented and involved testing particular interfacing and checking for feasibility of classification of email through ontologies. The challenge we faced was mainly to make C4.5 classification outputs to RDF and to give it to Jena, that is, interfacing two independent systems and creating a prototype that actually uses this information that flows from one system to another to get certain desired input. In our case, it was the classification of email.

With the default settings in Weka, all experiments were performed. Extensive experiments with different settings are applicable in Weka. Moreover, different algorithms which are not included in Weka can be tested. Also, experiments with various feature selection techniques should be compared. We implemented the dynamic ontologies as spam filter based on classification result. Then, this ontology is evolved and customized based on user's report when a user requests spam report. By creating a spam filter in the form of ontology, a filter will be user-customized, scalable, and modularized; so it can be embedded to many other systems for better performance. We need to more focus on misclassified legitimate email than misclassified spam email; so we do not have to lose legitimate emails even though we get some spam emails in mail box. Also, Overfitting can happen because the filter is learning, hence we consider this problem. Finally, currently many filters are dependent on different dataset and hence we need to develop more general filter, which can show a good performance on the most of dataset.

## Figures and Tables

**Algorithm 1:**
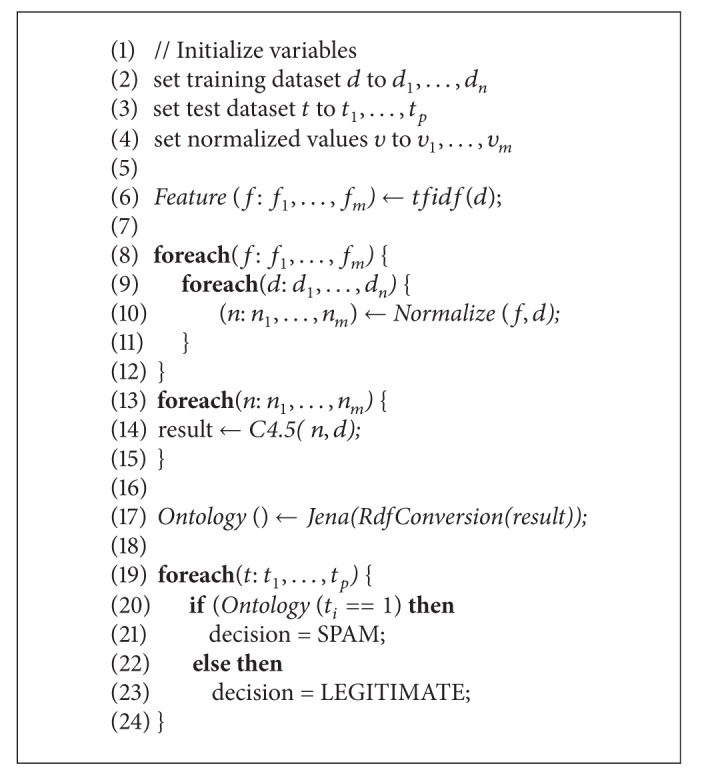
Global ontology filter pseudocode.

**Algorithm 2:**
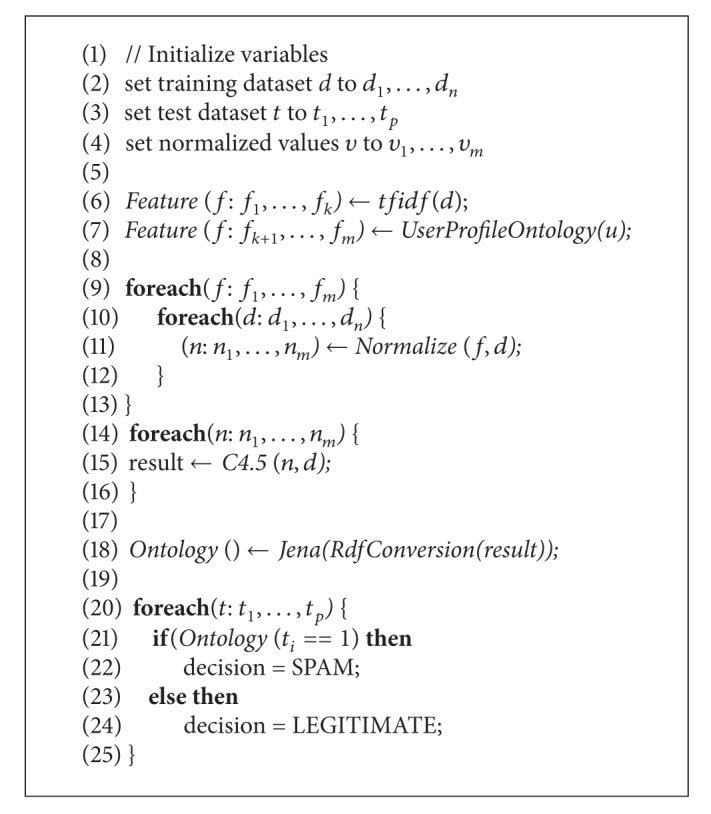
User-customized ontology filter pseudocode.

**Algorithm 3:**
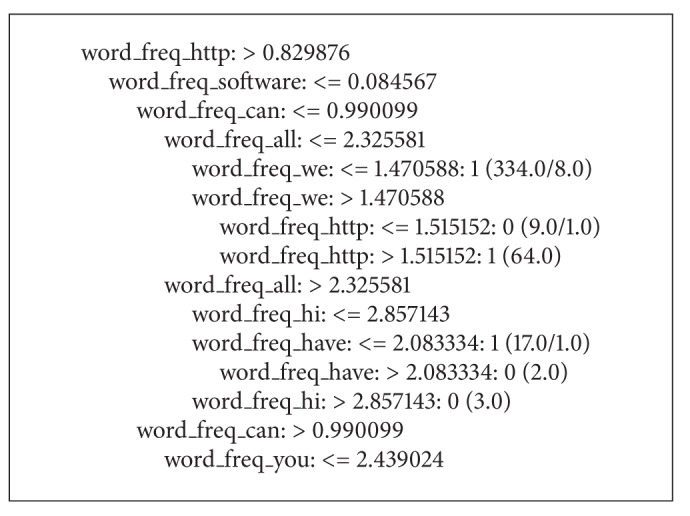
Part of C4.5 classification result.

**Algorithm 4:**
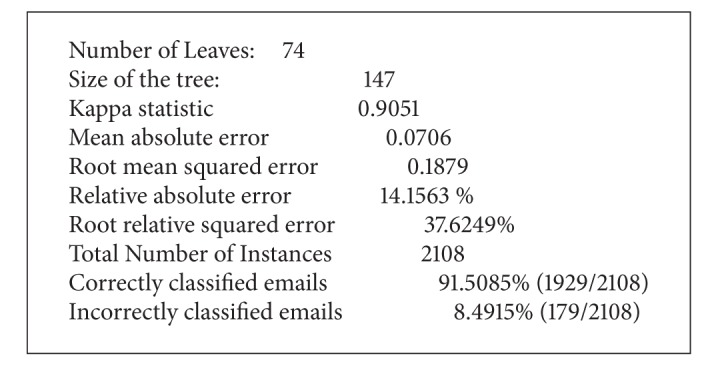
Summary of classification result.

**Table 1:** Classification result of training dataset.

Class	Precision	Recall
Spam	0.872	0.963
Nonspam	0.962	0.871

**Table 2:** Classification results of 200 user datasets.

Number of leaves	12
Size of tree	23

**Table 3:** Precision and recall of 200 user datasets.

Class	Precision	Recall
Spam	0.990	0.960
Legitimate	0.961	0.990

**Table 4:** Classification results of 400 user datasets.

Number of leaves	19
Size of tree	37

**Table 5:** Precision and recall of 400 user datasets.

Class	Precision	Recall
Spam	0.948	0.910
Legitimate	0.913	0.950

**Table 6:** Classification results of 600 user datasets.

Number of leaves	27
Size of tree	53

**Table 7:** Precision and recall of 600 user datasets.

Class	Precision	Recall
Spam	0.882	0.973
Legitimate	0.970	0.870

**Table 8:** Precision and recall of SPONGY system.

Class	Precision	Recall
Spam	0.931	0.977
Legitimate	0.978	0.934

**Table 9:** Results of SPONGY system.

	Global ontology	User ontology w/200	User ontology w/400	User ontology w/600
Spam (1008)	37	27	24	23
Legitimate (1100)	142	87	85	73
Correct classification	91.5085%	94.5920%	94.8292%	95.4459%

**Table 10:** Spam filter comparison.

	SpamEater Pro	CA Anti-Spam	ChoiceMail One	Spam killer	SPONGY
Block IP address	O		O		O
Block server	O		O	O	O
Block email address	O	O	O	O	O
Blacklist support	O				O
Allow IP address	O		O		O
Allow server	O		O	O	O
Allow email address	O	O	O	O	O
Individual user profile	O		O	O	O
Reporting capability			O	O	O
